# Salivary DJ-1 could be an indicator of Parkinson's disease progression

**DOI:** 10.3389/fnagi.2014.00102

**Published:** 2014-06-06

**Authors:** Wen-Yan Kang, Qiong Yang, Xu-Feng Jiang, Wei Chen, Lin-Yuan Zhang, Xiao-Ying Wang, Li-Na Zhang, Thomas J. Quinn, Jun Liu, Sheng-Di Chen

**Affiliations:** ^1^Department of Neurology, Institute of Neurology, Ruijin Hospital affiliated to Shanghai Jiaotong University School of MedicineShanghai, China; ^2^Department of Nuclear Medicine, Ruijin Hospital affiliated to Shanghai Jiaotong University School of MedicineShanghai, China; ^3^Department of Biostatistics, Shanghai Jiaotong University School of MedicineShanghai, China; ^4^Department of Radiation Oncology, Albert Einstein College of Medicine of Yeshiva UniversityBronx, NY, USA

**Keywords:** DJ-1, saliva, Parkinson's disease, dopamine transporter, SPECT

## Abstract

**Objective:** The goal of the current investigation was to explore whether salivary DJ-1 could be a potential biomarker for monitoring disease progression in Parkinson's disease (PD) by evaluating the association between salivary DJ-1 concentrations and nigrostriatal dopaminergic function.

**Methods:** First, in 74 patients with PD and 12 age-matched normal controls, single photon emission computed tomography (SPECT) imaging with labeled dopamine transporters (DAT) (^99m^Tc-TRODAT-1), which has been used for measuring DAT density in PD was prformed. Then, the DJ-1 level in their saliva was analyzed by quantitative and sensitive Luminex assay and compared to caudate or putamen DAT density. Finally, based on the above, our cross-section study was carried out in 376 research volunteers (285 patients with PD and 91 healthy controls) to measure salivary DJ-1 level.

**Results:** From our analysis, we found a correlation between salivary concentration of DJ-1 and putamen nucleus uptake of ^99m^Tc-TRODAT-1 in the PD group. Although salivary DJ-1 levels were not affected by UPDRS scores, gender, age, and pharmacotherapy, DJ-1 levels in H&Y 4 stage of PD were higher than those in H&Y 1-3 stage as well as those in healthy controls. Salivary DJ-1 also decreased significantly in mixed type PD patients compared to the tremor-dominant type (TDT) and akinetic-rigid dominant type (ARDT) PD patients.

**Conclusions:** According to the investigation in a large cohort, we reported for the first time the prognostic potential of the salivary DJ-1 as a biomarker for evaluating nigrostriatal dopaminergic function in PD.

## Introduction

Parkinson disease (PD), the second most common neurodegenerative disorder, affects approximately 1.6% of the population over the age of 65 (Wright Willis et al., 2010). About 70% of dopaminergic (DA) neurons in the substantia nigra are lost prior to the development of the characteristic motor symptoms of PD (Sherer, 2011). Consequently, an early diagnosis of PD is difficult, by virtue of the dependence upon clinical features. Therefore, the identification of useful biomarkers to make an early diagnosis, or for monitoring progression, of PD is needed (Jankovic, 2008; Haas et al., 2012).

Positron emission tomography (PET) and single photon emission computed tomography (SPECT) with radiotracer imaging in the presynaptic nigrostriatal dopaminergic system has enabled the study of nigrostriatal dopaminergic degeneration in PD patients, a technique with excellent reproducibility for improving clinical diagnosis, monitor disease progression, and evaluate the efficacy of putative neuroprotective therapies (Marek et al., 2001; Stoessl, 2012). ^99m^Tc-TRODAT-1, developed by Kung et al. (1997), has high affinity and selectivity for Dopamine transporters (DAT), which are located in dopaminergic nerve terminals and mediate dopamine reuptake. In addition to its clinical advantages, such as stability and low toxicity, ^99m^Tc-TRODAT-1 SPECT imaging has excellent test/retest reproducibility for longitudinal evaluation of nigrostriatal dopaminergic function in PD patients (Hwang et al., 2004).

In recent years, a number of biochemical molecules, such as α-synuclein, DJ-1, tau, etc., in cerebrospinal fluid (CSF) and blood have been studied as potential biomarkers of PD (Hong et al., 2010; Waragai et al., 2010), (Mollenhauer et al., 2006; Waragai et al., 2007). It has been reported that CSF DJ-1 levels in the early stage of PD were significantly higher than those in the advanced stage of PD and non-PD controls (Waragai et al., 2006). However, another study that included a larger cohort of subjects demonstrated that CSF DJ-1 levels were decreased in PD patients vs. controls (Hong et al., 2010). Moreover, it has also been shown that plasma DJ-1 levels in PD patients were higher than those in control patients (Waragai et al., 2007), although another study did not find a difference between PD and control patients (Maita et al., 2008). These conflicting results are likely due to the limited numbers of patients, lacking of an efficient detection method, or biofluid contamination.

Our collaborators have demonstrated that DJ-1 was present in human saliva in both PD patients and normal controls (NC) (Devic et al., 2011). However, there was no significant difference between the two groups. It is still not clear whether salivary DJ-1 could be a useful indicator to monitor the progression of PD. In the present study, we measured salivary levels of DJ-1, followed by analyzing its correlation with DAT, quantified by ^99m^Tc-TRODAT-1 SPECT. Secondly, this study analyzed a large cohort of subjects to further investigate salivary DJ-1 as a potential biomarker of PD.

## Materials and methods

### Participants

This study was approved by the Ethics Committee of Ruijin Hospital affiliated to Shanghai Jiaotong University School of Medicine. All subjects provided their written informed consent for the participation in the study and underwent a thorough evaluation by obtaining a medical history, physical history, laboratory tests, neurological examinations, and neuropsychological assessments performed by multiple rating scales, including UPDRS (Unified Parkinson's Disease Rating Scale), MMSE (the mini–mental state examination), the HAMD-17 (17-item Hamilton Rating Scale for Depression), SCOPA-AUT and REM Sleep Behavior Disorder scales. In addition, UPDRS motor scores were assessed when patients were in the “on” state. The diagnosis of PD patients was made by at least two senior movement disorder specialists in accordance with the UK PD Society Brain Bank Clinical Diagnostic Criteria of PD (Hughes et al., 1992). Recruited control subjects were from the Shanghai Wuliqiao community and were matched for age and sex with the former two disease groups, who had no history or any signs or symptoms suggesting Parkinson-plus syndrome, cognitive impairment, or other neurological diseases. Exclusion criteria included moderate or heavy cigarette smoking (more than 10 packs/year), alcohol use, and any psychotherapeutic drug use.

### Experimental procedures

#### Saliva sample collection and preparation

All saliva samples were collected as described previously with minor modifications based on the suggestion from our collaborator, Professor Jing Zhang at the University of Washington in Seattle, WA, USA (Devic et al., 2011). All collection procedures were performed between 9:00 and 11:00 am to avoid any potential confounding effects of circadian rhythm. The subjects were fasted 60 min prior to sample collection. Five minutes before the collection, each subject rinsed his mouths with water for 3 min to remove any excess tissue or debris. The subject was told to tilt his head forward to accumulate his saliva in the mouth and each sample was collected in a 15 ml pre-chilled vial and kept in the ice before further processing. Saliva samples were collected in a resting and unstimulated state (i.e., no food, chewing gum, etc.). When extracting the protein, Protease Inhibitor Cocktail (100 μl/1 ml of whole saliva, Cat# P2714, Sigma Aldrich, St. Louis, MO, USA) was added to the sample to minimize protein degradation, and the sample was vortexed repeatedly followed by two centrifugations. The initial centrifugation was at a low rate of 2600 × g for 15 min at 4°C. The precipitate was preserved and stored at −80°C. The supernatant was subject to a second centrifugation at a high rate of 15,000 × g for 15 min at 4°C. Again, the precipitate was preserved and stored at −80°C and the supernatant was then divided into 0.5 ml aliquots and stored at −80°C before analysis. The total salivary protein concentration was measured using a bicinchoninic acid (BCA) protein assay kit (Pierce, Rockford, IL, USA).

#### Luminex assay

***Coupling of primary antibody to magnetic COOH beads***. Magnetic COOH beads (Cat# MC10052-01, Bio-Rad, USA) were chemically coupled with a rabbit polyclonal PARK7 (DJ-1) antibody (Cat# Ab18257, Abcam, USA) with the amine coupling kit (Cat# 171-406001, Bio-Rad, USA) according to the manufacture's protocol (Figure S1). Briefly, 100 μl beads were activated with 10 μl EDC (1-ethyl-3-[3-dimethylaminopropyl], 50 mg/ml) and Sulfo-NHS (N-hydroxysulfosuccinimide, 50 mg/ml) in the ProteOn™ Amine Coupling Kit (Cat# 1762410, Bio-Rad, USA). Ten to Twenty μg of primary antibodies were added to the activated beads and incubated for 2 h. The coupled beads were re-suspended in 150 μl of storage buffer or an alternate storage buffer to complement the protein assay. Determination of the bead concentration was performed using a Coulter Z2 counter or a hemocytometer to validate the efficiency of the coupling reaction. The coupled beads were then stored at 4°C and covered with aluminum foil.

***Magnetic bead-based luminex assay***. Salivary DJ-1 levels were measured using established Luminex assays as described previously, with minor modifications (Devic et al., 2011) Briefly, 50 μl of capture antibody-coupled beads (about 2500 beads per well) were added to 96 well Bio-Plex Pro Flat Bottom Plates (Cat# 171025001, Bio-Rad, USA) and washed twice using the reagent kit (Cat# 171304071, Bio-Rad, USA). Then, 50 μl diluent recombinant human DJ-1 (Covance, USA) were used as standards, and saliva samples, diluted in equal sample dilution, were loaded on the magnetic plate with incubation for 2 h at 1000 rpm on a plate shaker at room temperature in the dark, followed by washing the plate three times in succession. Subsequently, detecting antibodies (0.1 μg/ml, biotinylated anti-human DJ-1 antibody, Cat#BAF3995, R&D systems, USA) were added at 50 μl per well on a rotator at room temperature for 60 min followed by washing three times, and the streptavidin-PE was diluted with assay buffer in the reagent kit for 30 min. The plate was then washed three times and each well received 125 μl assay buffer, followed by shaking for 3 min and then read via Liquichip Luminex 200TM. The concentration of samples was calculated by comparison to a best-fit standard curve using a sigmoidal 5-parameter logistic equation (Figures S1, S2). The salivary DJ-1 signal-to-background ratio was 64. The recovery rate was close to 81% and coefficient of variation (CV) in duplicate was less than 20%. Finally, the Luminex assay demonstrated low day-to-day as well as plate-to-plate signal variability (<10%), with high signal reproducibility (Figures S3, S4).

#### SPECT imaging

All subjects were prohibited from any pharmacotherapy affecting the experimental curative effect observation (particularly dopamine-specific drugs) within the 48 h prior to receiving an intravenous injection of 2 ml containing 20 mCi of ^99m^Tc-TRODAT-1 in an antecubital peripheral vein. The binding to DAT was assessed with SPECT imaging 2 h after injection. ^99m^Tc-TRODAT-1 was prepared from a pre-formulated lyophilized kit provided by Jiangsu Nuclear Medicine Institute (WuXi city, China). Subjects were put on the scanning bed in the supine position with a head holder to avoid motion artifact. All images were acquired via a double-headed gamma camera (Simens, Symbia T16). The energy window was 140 ± 14 keV, and a matrix of 128 × 128 in circular orbit with step and shoot movements of 64 steps used for each head, with a diameter and degree of rotation of 30 cm and 360°, respectively. Acquisition time for the projection was 30 s, with a zoom of 1.45 and a slice thickness of 3 mm. Each patient also underwent brain MRI (1.5 T, Siemens) with 3 mm cuts at the level of the basal ganglia. The MRI study was performed to establish a reference for the determination of regions of interest (ROIs) for the uptake of ^99m^Tc-TRODAT-1 visualized with SPECT.

#### Imaging analyses

Based on individual MRI images, the (ROIs, 776 ± 33 mm^2^) were placed over the left and right striatum divided into the caudate nucleus and putamen. The reference background ROI (2864 ± 60 mm^2^) was placed on the cerebellum of the same summed image. Two outcome measures were computed. The specific striatal uptake was measured 2 h after injection and was calculated for both the left and right striatum as the following: binding potential of the non-displaceable radioligand (BP_ND_) = ST-CB/CB, where ST, striatum (caudate and putamen) and CB, cerebellum, and the results were then averaged.

### Statistical analysis

Statistical analyses were performed using SAS version 9.2 (SAS Institute, Cary, NC) and GraphPad Prism for Windows version 5.0 (GraphPad Software, San Diego, CA, USA). To compare enumeration data among groups, chi-square test or Fisher's exact test was used. We analyzed the data by *t*-test or One-Way analysis of variance (ANOVA) if the data was normally distributed. Non-parametric Kruskal–Wallis ANOVA was used, followed by a Mann–Whitney *U*-test for continuous variables if data was not normally distributed. Additionally, to determine whether a relationship among variables was present, we used an analysis of covariance (ANCOVA) model after rank transformation of the non-normal data to control for potential contributions secondary to outliers. The analyses were done with and without adjustment for potential confounding of the baseline variables—i.e., age, gender. To determine whether a relationship among variables was present, we obtained the non-parametric spearman correlation coefficients. All values were expressed as mean ± SD. A two-tailed *p* value < 0.05 was defined as a statistically significant difference.

## Results

### Striatal dopaminergic function and salivary DJ-1 levels

**Figure 1:**
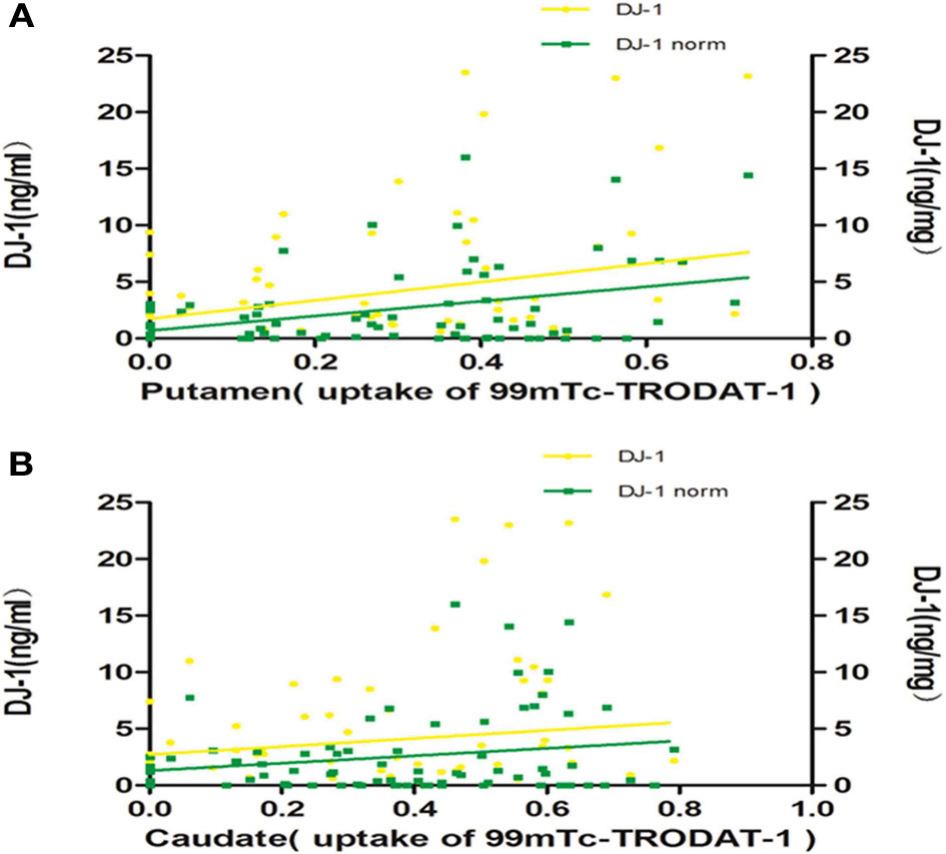
**Salivary DJ-1 levels and caudate, putamen uptake of ^99m^Tc-TRODAT-1**. The uptake of ^99m^Tc-TRODAT-1 was measured by SPECT and DJ-1 levels in saliva were measured by Luminex assay in 74 PD patients. **(A)** Non-parametric spearman's correlation analysis revealed that salivary DJ-1 levels were weakly associated with putamen nucleus uptake of ^99m^Tc-TRODAT-1 in PD patients (*p* = 0.20, *R* = 0.151, *p* = 0.026, *R* = 0.258 when normalized). **(B)** Statistical analysis revealed that salivary DJ-1 levels did not correlate with caudate nucleus uptake of ^99m^Tc-TRODAT-1 in PD patients whether normalized (*p* = 0.72, *R* = 0.043) or not (*p* = 0.73, *R* = −0.041).

Although there was no statistically significant difference between the levels of salivary DJ-1 and UPDRS scores in a previous study (Devic et al., 2011), the results suggested that there might be a “floor” effect when using UPDRS motor scores. Consequently, we adopted ^99m^Tc-TRODAT-1 SPECT, a method that has already proved its important role in the diagnosis and progression of PD, to evaluate salivary DJ-1 as novel potential biomarker reflecting nigrostriatal dopaminergic function. As a pilot study, we recruited 12 healthy controls and 74 PD patients, who were matched for age and sex in the two groups (Table 1). A non-parametric spearman correlation analyses revealed that salivary DJ-1 levels slightly correlated with putamen nucleus uptake of ^99m^Tc-TRODAT-1 in PD patients (Figure 1A) but not within the caudate nucleus (Figure 1B). No correlation between salivary DJ-1 and the uptake of ^99m^Tc-TRODAT-1 in either the caudate nucleus or putamen in the control group was identified (data not shown), with the potential confounding variables of sex and age controlled for.

**Table 1:** **Summary of demographics and mean regional caudate nucleus, putamen ^99m^Tc-TRODAT-1 BP_ND_ values in PD patients and control subjects**.

	**Patients**	**Controls**
Age	61.8 ± 7.8	55.5 ± 6.11
M:F	50/24	6/6
Duration (years)	4.36 ± 3.59	
UPDRS III	17.63 ± 11.69	
Average caudate	0.39 ± 0.21^**^	1.17 ± 0.40
Average putamen	0.29 ± 0.20^**^	1.11 ± 0.35

Values are means ± SD,

** p < 0.01 vs. controls, a non-parametric Mann–Whitney U-test for variables was used for comparison. (BP_ND_, Non-displaceable radioligand; DAT, Dopamine transporters; UPDRS, Unified Parkinson's Disease Rating Scale).

### Salivary DJ-1 in healthy controls and patients with PD in larger cohort

**Figure 2:**
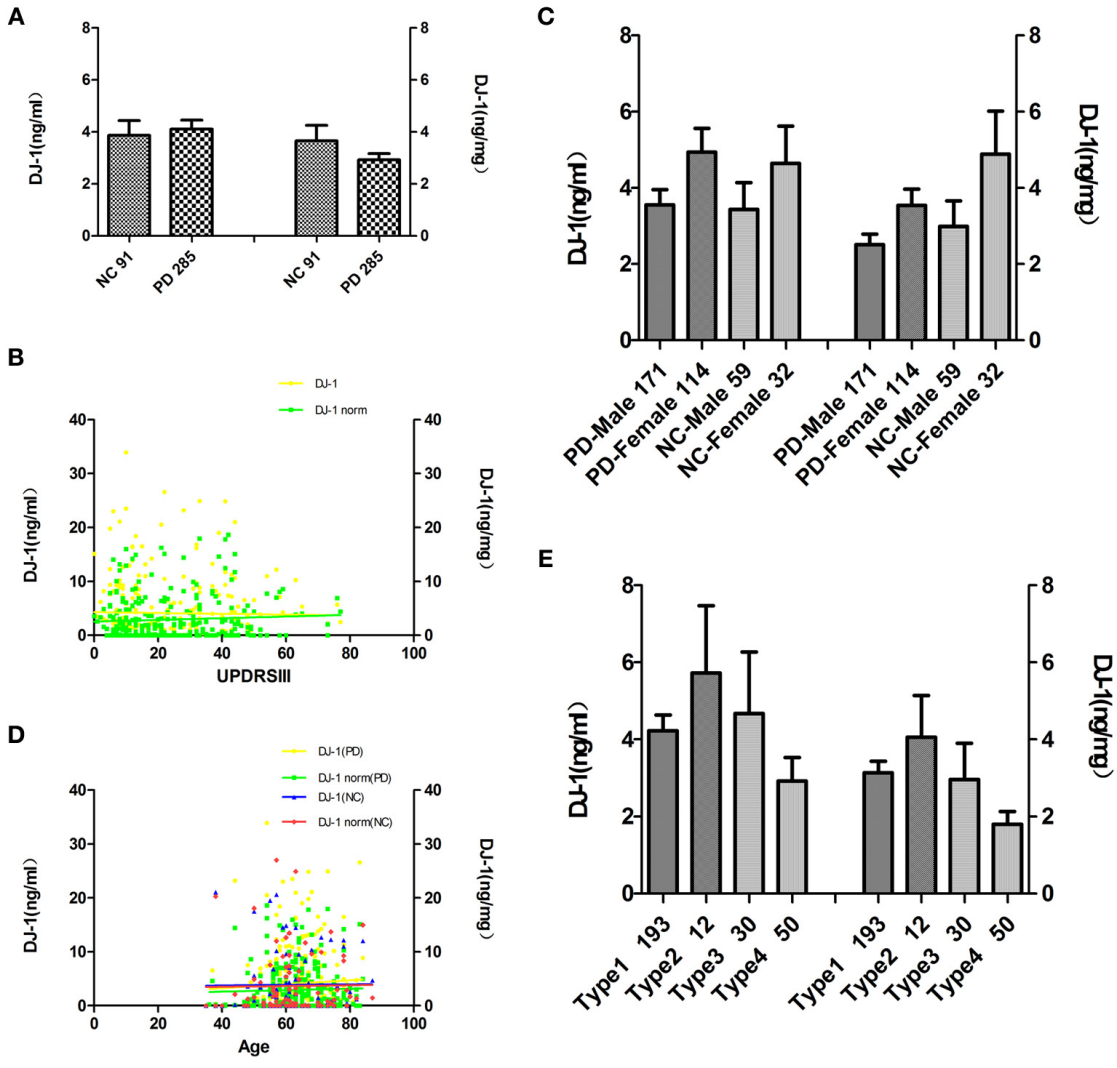
**Analysis of salivary DJ-1 in healthy controls and patients with PD in a large cohort**. Salivary DJ-1 levels were measured in 91 individual NC and 285 PD samples by Luminex assay. **(A)** Quantitative Luminex analysis of salivary DJ-1 levels in patients with PD and NC by the non-parametric Mann-Whitney *U*-test, before and after controlling for potential contributions secondary to outliers (e.g., age, sex), and no significant differences were found (*p* = 0.72, *p* = 0.84 when normalized). **(B)** Salivary DJ-1 correlated with disease severity as measured by UPDRS motor scores was analyzed by the non-parametric spearman's correlation analysis, no statistically significance was achieved (*p* = 0.92, *R* = 0.006; *p* = 0.44, *R* = 0.05 when normalized). **(C)** Salivary DJ-1 levels did not correlate with gender both in individual Parkinson's disease and control group (*p* = 0.21, *p* = 0.054 when normalized in PD group, *p* = 0.20, *p* = 0.17 when normalized in the NC group). **(D)** Salivary DJ-1 levels did not correlate with age both in individual Parkinson's disease and control group (*p* = 0.32, *p* = 0.81 when normalized in PD group; *p* = 0.54, *p* = 0.49 when normalized in the NC group). **(E)** There is no significant difference among the different drug treatment groups (**Type1, Type2, Type3, Type4**) even in the same disease stage (data not shown), whether normalized (*p* = 0.086) or not (*p* = 0.25). Data shown are mean ± SD. A two tail *p*-value <0.05 was defined as statistically significant.

Since the above results demonstrated that salivary DJ-1 correlated with striatal dopaminergic function, indicating a potential value in the diagnosis of PD, we sought to confirm this finding in a larger cohort of patients. To accomplish this, 285 PD patients (171 men, 114 women) were recruited for the study. The demographic data is listed in Table 2, of which the amount of DJ-1 in different groups was quantified by Luminex assay. The results demonstrated that there was no statistically significant difference in DJ-1 levels in the PD group compared to the NC group (Figure 2A), which was consistent with a previous report (Devic et al., 2011). In addition, we evaluated the correlation between DJ-1 levels and UPDRS scores of 285 PD patients, and found that the correlation was of no statistical significance, even after normalization (Figure 2B). Since there was no difference in salivary DJ-1 levels between PD and NC, we further evaluated whether or not gender, age, or therapeutics affected DJ-1 levels. We found no correlation between gender, age and DJ-1 levels in PD and NC groups (Figures 2C,D). Furthermore, no correlation was found between therapeutic use and DJ-1 levels, whether normalized or not (Figure 2E). The above results indicated that salivary DJ-1 was not affected by gender, age, or pharmacotherapy.

**Table 2:** **Summary of demographics and salivary DJ-1 levels in a large cohort**.

	**Patients**	**Control**
Number of Cases	285	91
Age (years)	63.34 ± 9.11	61.59 ± 10.61
M:F	171/114	59/32
UPDRS III	23.8 ± 15.7	
Cases of Drug Treatment^a^	193:12:30:50	
Salivary total protein (mg/ml)	1.55 ± 1.07	1.34 ± 0.95
DJ-1 (ng/ml)	4.11 ± 5.88	3.86 ± 5.44
DJ-1 normal (ng/mg)	2.92 ± 4.02	3.66 ± 5.66

Values are means ± SD.

a Number of patients with Parkinson's disease who were treated with carbidopa/levodopa alone or together with other anti-parkinsonism drugs (***Type1***) vs. those treated with dopamine agonists but not levodopa (***Type2***) vs. those treated with other anti-Parkinson's disease medications (e.g., monoamine oxidase B inhibitors and amantadine) only (***Type3***) vs. those not treated with any anti-parkinsonism drugs (no Parkinson's disease medication) when the saliva samples were obtained (***Type4***).

### Alteration of salivary DJ-1 in different stages and clinical subtypes of PD

**Figure 3:**
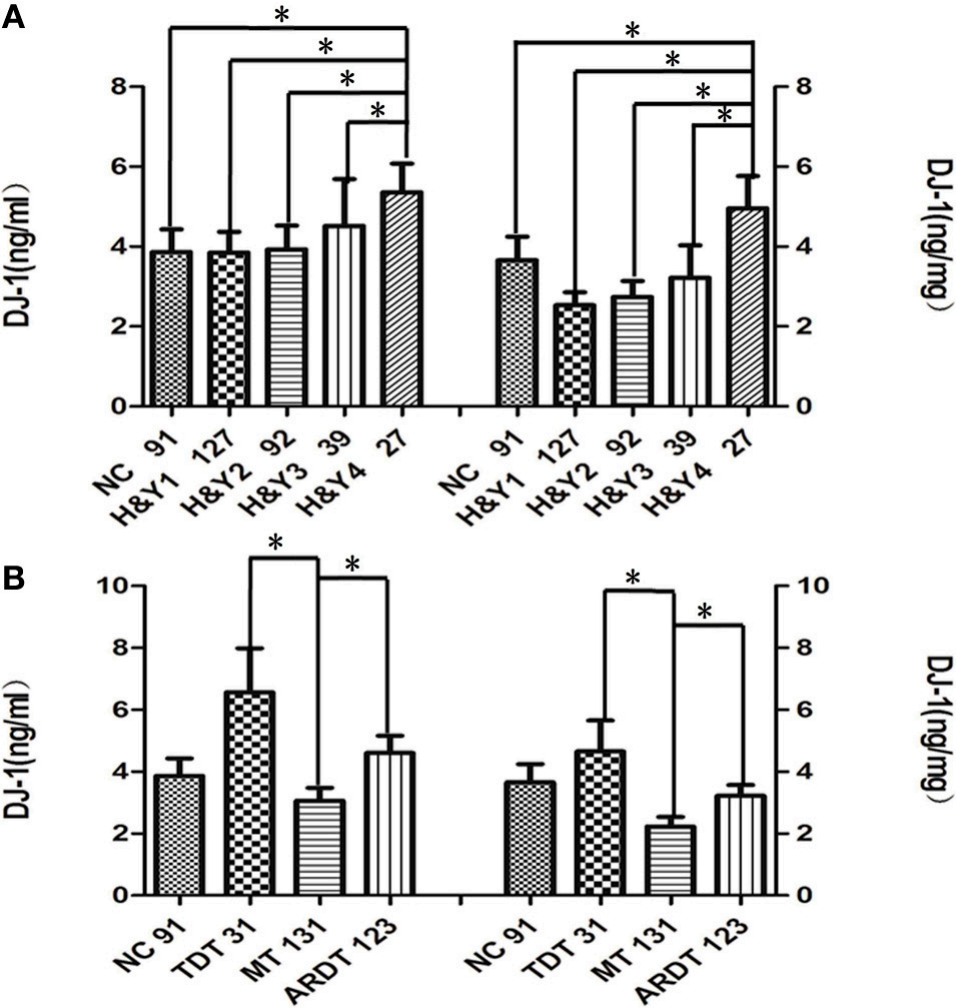
**Alteration of salivary DJ-1 in different stages and clinical subtypes of PD. (A)** The PD group was divided into four subgroups according to the Hoehn & Yahr staging, and statistical analysis of salivary DJ-1 levels in the different disease stages performed: the DJ-1 levels increased in H&Y-4 stages of PD compared to the other three groups and NC groups (H&Y-1 vs. H&Y-4 *p* = 0.001, H&Y-2 vs. H&Y-4 *p* = 0.003, H&Y-3 vs. H&Y-4 *p* = 0.014, NC vs. H&Y-4 *p* = 0.005, H&Y-1 vs. H&Y-4 *p* = 0.0002, H&Y-2 vs. H&Y-4 *p* = 0.001, H&Y-3 vs. H&Y-4 *p* = 0.014, NC vs. H&Y-4 *p* = 0.005 when normalized). **(B)** PD patients were classified as tremor-dominant type (TDT), if the tremor score was at least twice the non-tremor score. The patient was classified as akinetic-rigid dominant type (ARDT) if the non-tremor score was at least twice the tremor score. The remaining patients, in whom the tremor and non-tremor scores differed by less than a factor of 2, were classified as mixed type (MT). From the analysis, MT patients had lower DJ-1 levels than the TDT group (*p* = 0.02, *p* = 0.054 when normalized) and ARDT group (*p* = 0.016, *p* = 0.032 when normalized) when PD patients were divided into tremor dominant, akinetic, and mixed subtypes. Data shown are mean ± SD. A two tail *p*-value < 0.05 was defined as statistically significant.

Since salivary DJ-1 did not correlate with UPDRS scores, we sought to stratify salivary DJ-1 levels by disease stage or clinical subtype. To determine whether salivary DJ-1 levels were altered during the course of disease, PD patients were divided into different groups according to Hoehn & Yahr (H&Y) stages. After adjustment for age and gender, the results revealed that salivary DJ-1 levels in H&Y-4 stage of PD were higher than those in H&Y 1-3 stage (Figure 3A). Moreover, DJ-1 levels in H&Y-4 stage were higher than those in healthy controls (Figure 3A). These results suggested that salivary DJ-1 might be a potential biomarker for monitoring disease progression.

In addition, previous studies have shown that different subtypes of PD may have different disease progression (Kumru et al., 2007). Therefore, we further investigated whether salivary DJ-1 levels were altered in different clinical subtypes of PD, including tremor dominant type (TDT), akinetic-rigid dominant type (ARDT), and mixed type (MT) (Romenets et al., 2012). In our study, 285 PD patients were divided into the above three subtypes, according to the criteria developed by Carsten Eggers et al. (2011). After adjustment for age and gender, we found that salivary DJ-1 decreased significantly in MT patients compared to TDT and ARDT patients (Figure 3B). However, there was no significant difference between ARDT patients and other groups.

## Discussion

To our knowledge, this study is the first to measure salivary DJ-1 in a large cohort of PD patients and healthy individuals. The present investigation has resulted in several major advances. Firstly, although our results demonstrated that DJ-1 levels in the saliva were not changed significantly in PD patients compared with NC, salivary DJ-1 levels were weakly associated with putamen uptake of ^99m^Tc-TRODAT-1 in PD patients. Secondly, several factors critical to DJ-1 levels in saliva were determined, including the influence of gender, age, and drug effects. Lastly, we observed a significant difference in salivary DJ-1 levels in PD patients based on different disease stages and clinical subtypes.

Saliva is an extraordinary medium in terms of research and diagnostic possibilities (Zhang et al., 2012). Discovery of salivary biomarkers offers an easy, inexpensive, safe, and non-invasive alternative to blood/CSF for the detection of disease. Although great progress has been made in the research on salivary biomarkers (Bonne and Wong, 2012), there are limited studies in PD patients. DJ-1 is detectable in both cellular (pellet) and acellular (supernatant) components of saliva, and multiple potential sources may contribute to DJ-1 in saliva. The human submandibular gland which is linked to the central nervous system, as the main source of the saliva, has recently been shown to be involved by synucleinopathy in the early stages of PD. Cheek epithelium, which forms the majority of the cellular component of saliva, could be another potential sources of salivary DJ-1 (Stewart et al., 2014). Devic's study has demonstrated that DJ-1 was detectable in human saliva, suggesting that saliva could be a potentially important diagnostic sample source for PD diagnosis or monitoring (Devic et al., 2011), (Wang et al., 2011). To further evaluate the clinical value of salivary DJ-1 for PD diagnosis, we conducted a large cohort study with a total of 376 individuals.

In a previous study (Devic et al., 2011), DJ-1 levels in saliva were estimated to be 185.7 ± 339 ng/ml in PD patients and 128 ± 116.4 ng/ml in controls. In contrast, our findings showed that the concentration of salivary DJ-1 in PD and controls were 4.11 ± 5.87 and 3.86 ± 5.44 ng/ml, respectively. The discrepancy between the two studies could be due to several major differences between the two investigations. The different capture antibodies might detect different species of DJ-1. Also, in the current investigation, a much larger cohort (91 NC vs. 285 patients with PD) was studied, thus allowing adequate power to resolve several potential factors which may significantly affect salivary DJ-1 levels.

In our study, we found a weak correlation between salivary DJ-1 levels with putamen uptake of ^99m^Tc-TRODAT-1 in 74 PD patients, which was inconsistent with the previous study, in which there was no correlation between CSF DJ-1 and PET measures of striatal DA function in total of 26 LRRK2 mutation carriers (Shi et al., 2012). The different capture antibodies and patients with different genotype may contribute to the difference.

In the present study, no relationship between DJ-1 levels and total UPDRS scores was identified. Nevertheless, we found that salivary DJ-1 levels in the advanced stage of PD were significantly higher than those in the early stage of PD, which is similar to plasma DJ-1 results (Waragai et al., 2007). As the disease progresses, the DJ-1 in saliva and plasma are increased, reflecting the involved process of increasing oxidative stress in advancing stages of PD (Winkler-Stuck et al., 2005; Waragai et al., 2007; Wang et al., 2011).

In this study, we studied potential confounders that might affect the generation of salivary DJ-1. Age and gender dependence were analyzed in both PD and control samples. Of note, we found no association of DJ-1 levels with gender or age, which is inconsistant with a recent report that found DJ-1 levels in the CSF increased significantly as a function of age (Hong et al., 2010). We also collected information on drug treatment and analyzed the potential effects of pharmacotherapy on salivary DJ-1 level. Neither did we find that DJ-1 values in the different groups were any statistically significant difference. Pharmacotherapy did not affect salivary DJ-1 levels either. However, it remains unknown whether the activity of DJ-1 generation is influenced by other factors. For example, it is important to study the effects of drug dose on salivary DJ-1 levels, however, similar to the research on CSF DJ-1 mentioned before, it is rather difficult to obtain accurate cumulative dosage of drug treatment in an investigation due to alterations of both the types of medication and the dosage of all drugs taken over time (Hong et al., 2010).

PD can be classified into different clinical subtypes based on clinical features and pathogenic mechanisms. The different subtypes exhibit distinct disease courses, drug therapy responses and prognosis (Shih et al., 2007; Eggers et al., 2011). For instance, motor function deteriorates more rapidly in ARDT than TDT cases (Kumru et al., 2007). The underlying mechanisms for the different subtypes are not yet clear. In our study, DJ-1 levels in saliva in the mixed PD subtype, which one would expect to be the intermediate subtype between ARDT and TDT subtypes, decreased significantly compared to both the TDT and ARDT patients. And the fact that the smaller number of patients included in TDT groups of PD may explain the result. In spite of it, our study indicated that different clinical subtypes might have different mechanisms for disease progression. This finding may help improve strategic planning of therapy or providing a predictor for disease prognosis.

In the scope of this study, we have not evaluated the salivary DJ-1 levels in other parkinsonian disorders whose symptoms may overlap with sporadic PD clinically, such as ET, PSP, and MSA, etc. Further studies with disease control for important confounding factors are necessary to determine whether salivary DJ-1 could differentiate PD from those related disorders.

In summary, our data indicated that DJ-1 in saliva as a potential diagnostic biomarker is not for distinguishing PD patients from healthy controls in a large cohort. Neither was it influenced by gender, age, and pharmacotherapy. The DJ-1 levels in saliva were well correlated with the different disease stages, thus could be useful for evaluating disease progression and different clinical subtypes of PD.

## Author contributions

Sheng-Di Chen and Jun Liu conceived and supervised the project. Sheng-Di Chen and Jun Liu are responsible for subjects' recruitment and experimental design and execution. Wen-Yan Kang, Jun Liu and Thomas J. Quinn were drafted the manuscript. Wen-Yan Kang, Qiong Yang, Wei Chen and Xiao-Ying Wang were responsible for patient characterization and sample collection. Xu-Feng Jiang was responsible for SPECT analysis. Qiong Yang, Wen-Yan Kang and Lin-Yuan Zhang worked on sample handling and Luminex assays. Wen-Yan Kang and Li-Na Zhang worked on data management and statistical analyses. All authors critically reviewed the manuscript.

## Study funding

This work was supported by grants from the National Program of Basic Research (2010CB945200, 2011CB504104) of China, National Natural Science Fund (81071024, 81171202, 30870879 and 81228007), Shanghai Shuguang Program (11SG20), Shanghai Key Project of Basic Science Research (10411954500), and the Fifth National Undergraduate Student Innovating Program (2011015).

### Conflict of interest statement

The study was approved by the Ethics Committee of Ruijin Hospital affiliated to Shanghai Jiaotong University School of Medicine. The authors declare that the research was conducted in the absence of any commercial or financial relationships that could be construed as a potential conflict of interest.

## Abbreviations

PD: Parkinson's disease
CSF: Cerebrospinal Fluids
UPDRS: Unified Parkinson's Disease Rating Scale
MMSE: The mini–mental state examination
HAMD-17: The 17-item Hamilton Rating Scale for Depression
NMS: Non-Motor Symptom
TDT: Tremor Dominant Type
ARDT: Akinetic-Rigid Dominant Type
MT: Mixed Type
ST: Striatum
CB: Cerebellum
BP_*ND*_: Non-displaceable radioligand
DAT: Dopamine transporters
SPECT: Single photon emission computed tomography
PET: Positron emission tomography
EDC: 1-Ethyl-3-[3-dimethylaminopropyl
Sulfo-NHS: N-hydroxysulfosuccinimide
MP: Methylphenidate.
