# Supplementary material for: Salivary DJ-1 could be an indicator of Parkinson's disease progression
Source: Front Aging Neurosci. 2014 Jun 6;6:102. doi: 10.3389/fnagi.2014.00102 (PMC4047660; doi:10.3389/fnagi.2014.00102)
Supplement: Supplementary file 1 [file DataSheet1.DOC]

**Salivary DJ-1 could be an indicator of Parkinson’s disease progression**

Wen-Yan Kang^1,†^, Qiong Yang^1,†^, Xu-Feng Jiang^2,†^, Wei Chen^1^, Lin-Yuan Zhang^1^, Xiao-Ying Wang^1^, Li Na Zhang^3^, Thomas J. Quinn^4^, Jun Liu^1^,*^*^*, Sheng-Di Chen^1^,*^*^*

^1^ Department of Neurology & Institute of Neurology, Ruijin Hospital affiliated to Shanghai Jiaotong University School of Medicine, Shanghai, 200025, China

^2^ Department of Nuclear Medicine, Ruijin Hospital affiliated to Shanghai Jiaotong University School of Medicine, Shanghai, 200025, China

^3^ Department of Biostatistics, Shanghai Jiaotong University School of Medicine, Shanghai, 200025, China

^4^ Department of Radiation Oncology, Albert Einstein College of Medicine of Yeshiva University, NY, 10461, USA

† These authors contributed equally to this work

*** Corresponding authors:

Sheng-Di Chen or Jun Liu

Department of Neurology & Institute of Neurology, Ruijin Hospital affiliated to Shanghai Jiaotong University School of Medicine, Shanghai, 200025, China

Telephone: 86-21-6445-4473

Fax: 86-21-6445-4473

E-mail: chen_sd@medmail.com.cn

jly0520@hotmail.com

**Figure S1. Sensitivity of the established DJ-1 Luminex assay**


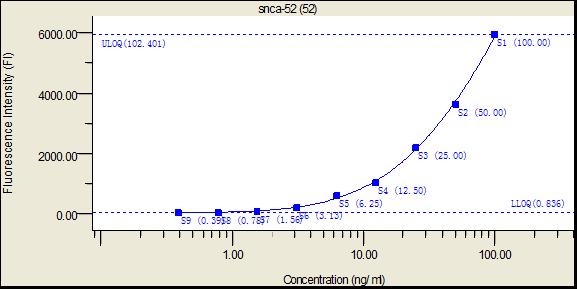


To establish DJ-1 Luminex assay, multiple antibody pairs were tested. After multiple antibodies tested, antibodies AB-DJ/RD-B (Cat#AB-18257, Abcam, USA/Cat# BAF3995, R&D, USA) gave the most consistent results and appeared to be more sensitive. So AB-DJ/RD-B was used in future analyses. Quantification was accomplished by incubating with streptavidin-conjugated fluorochrome-phycoerythrin and the sensitivity of the established DJ-1 Luminex ranged as low as 2.275 pg/ml of human DJ-1.

**Figure S2.** **Specificity of the established DJ-1 Luminex assay**

DJ-1 levels were determined by the Luminex assay in 50μg cerebral cortex extracts from DJ-1-null (KO) and wild-type (WT) mice, 50μg human cerebral cortex extract (human brain), 25μl un-concentrated pooled native human brain and 25μl human saliva.

**Figure S3.** **Characterization of saliva DJ-1 Luminex assay**

Identical aliquots of cell-free, native, previously unfrozen pooled saliva and thawed samples that were frozen at –80℃for 3 months were processed in parallel under the indicated conditions, and DJ-1 levels were determined by the Luminex assay. Compared with unfrozen samples, DJ-1 concentration in thawed samples changed little. T/C twice: freezing and thaw twice.

**Figure S4. The recovery rate of saliva DJ-1 Luminex assay**

DJ-1 concentrations were measured in un-spiked saliva samples (native diluted with sample dilution) and samples spiked with human recombinant DJ-1 proteins at indicated concentrations. The recovery rate was nearly 81%.
